# Differences in cancer incidence by broad ethnic group in England, 2013–2017

**DOI:** 10.1038/s41416-022-01718-5

**Published:** 2022-03-02

**Authors:** Christine Delon, Katrina F. Brown, Nick W. S. Payne, Yannis Kotrotsios, Sally Vernon, Jon Shelton

**Affiliations:** 1 Cancer Intelligence Team, Cancer Research UK, 2 Redman Place, London, E20 1JQ UK; 2 National Disease Registration Service, NHS Digital, 7 and 8 Wellington Place, Leeds, LS1 4AP UK

**Keywords:** Cancer epidemiology, Epidemiology

## Abstract

**Background:**

Cancer incidence variation between population groups can inform public health and cancer services. Previous studies have shown cancer incidence rates vary by ethnic group in England. Since their publication, the completeness of ethnicity recording in cancer data has improved, and relevant inequalities (e.g. risk factor prevalence and healthcare access) may have changed.

**Methods:**

Age-standardised incidence rates were calculated for Asian, Black, Mixed/Multiple and White ethnic groups in England in 2013–2017, using almost 3 million diagnoses across 31 cancer sites. Rate ratios were calculated with the White ethnic group as reference. Sensitivity analyses used imputed ethnicity for cases with missing data and perturbed population estimates.

**Results:**

Incidence rates for most cancer sites and ethnic group and sex combinations were lower in non-White minority ethnic groups compared with the corresponding White group, with particularly low rate ratios (below 0.5) for melanoma skin cancer and some smoking-related cancers (lung, bladder and oesophageal cancers). Exceptions included prostate cancer (2.1 times higher in males of Black ethnicity), myeloma (2.7–3.0 times higher in people of Black ethnicity), several gastrointestinal cancers (1.1–1.9 times higher in people of Black ethnicity, 1.4–2.2 times higher in people of Asian ethnicity), Hodgkin lymphoma (1.1 times higher in males of Asian ethnicity, 1.3 times higher in males of Black ethnicity) and thyroid cancers (1.4 times higher in people of Asian ethnicity, 1.2 times higher in people of Black ethnicity). Sensitivity analyses did not materially alter these results (rate ratios changed by a maximum of 12 percentage points, the direction and significance of results were unchanged in all but two cancer site/sex/ethnic group combinations).

**Conclusions:**

People of non-White minority ethnicity in England generally have lower cancer risk than the White population, though there are a number of notable exceptions. These results should galvanise efforts to better understand the reasons for this variation, and the possible impact on cancer services, patient experiences and outcomes.

## Introduction

Cancer incidence is markedly higher in majority White ethnicity world regions, such as Europe, North America and Oceania, compared with majority non-White ethnicity regions, such as Africa and Asia [1]. Although variation in data quality certainly contributes to these global differences [1], evidence from within countries with high-quality data also supports the existence of associations between ethnic groups and cancer incidence [2–11].

Understanding variation in cancer incidence between ethnic groups can inform public health measures to reduce inequality; for example, higher cancer incidence may reflect differential access to, or uptake of, screening [12–14], stop smoking services [15, 16], weight management services [17–19] or human papillomavirus (HPV) immunisation. It can also inform cancer service planning, to ensure the system is set up to support all affected groups; for example addressing factors like language barriers or unmet cultural/religious requirements, which may underpin differences between ethnic groups in routes to diagnosis [20], stage at presentation [21] and type of treatment received [22–24]. While cancer survival could be higher in non-White minority ethnic groups compared with the White ethnic group (although the evidence remains sparse) [2, 24–27], patients from non-White minority ethnic groups rate their overall care less favourably than White patients, and feel insufficiently involved in decisions about their care and treatment [28, 29].

Previous analyses of cancer incidence by ethnic group in the UK have found ethnic variation in the incidence of many cancer sites, with rates often lower in non-White minority ethnic groups compared with the White ethnic group, though this varies between and within cancer sites and individual ethnic groups [2, 9, 11, 24–26, 30, 31]. However for some cancer site/ethnic group combinations, such analyses have not been published to date, and for those cancer sites which have been analysed, there has been a degree of uncertainty because of limitations in the source data. Evidence on cancer incidence variation by ethnic group in the UK has various shortcomings including incomplete ethnicity recording in routinely collected hospital data and a lack of reliable population data by ethnic group. In previous analyses, ethnicity data were missing for around a quarter of cancer cases, and these missing values were imputed based on the observed data or hypothetical scenarios e.g. non-White minority ethnic groups being over- or under-represented in the missing data [2, 10]. For a number of cancer sites those imputation scenarios generated completely opposing results. For example, for all cancers combined in males, age-standardised incidence rates were significantly lower in the Black ethnic group compared with the White ethnic group if all the missing-ethnicity cases were assumed to be White. But if the missing-ethnicity cases were assumed to be distributed across ethnic groups in the same proportions as in the observed data, rates were significantly higher in the Black ethnic group compared with the White ethnic group [2]. This potential impact of missing ethnicity in cancer data, combined with the population estimates used having been found to be inconsistent with subsequent census data [32]; means the veracity of these previous results remains uncertain. Further, most existing analyses are over a decade old and may no longer reflect current levels of risk factor prevalence, screening uptake etc, for which trends may vary by ethnic group [33–36].

Although population data by ethnicity are still only estimated based on the decennial census, and major differences from previous results are not anticipated, an updated analysis of cancer incidence by broad ethnic group in England is warranted given the time elapsed since previous analyses and improvement in data completeness for ethnicity in England healthcare records from 2012 onwards. An evaluation of the impact of potential errors in population estimates, using the latest estimates produced in 2018, will be an integral part of this work. The aims of this paper are: first, to provide up-to-date case numbers and incidence rates for a wide range of cancer sites in broad ethnic groups in England; second, to use better-quality data to corroborate or refute the existing body of evidence on cancer incidence by broad ethnic group in England; and third, to examine whether feasible margins of error around the input data would materially change the results.

## Materials and methods

### Main analysis

The number of cancer cases by broad ethnic group, sex, 5-year age band and cancer site was obtained for England, 2013–2017, from Public Health England (PHE). The broad ethnic groups were: Bangladeshi, Chinese, Indian, Pakistani, any other Asian background (hereafter ‘Asian’), African, Caribbean, any other Black background (‘Black’), Mixed or Multiple ethnic groups (‘Mixed/Multiple’), Other ethnic groups (‘Other’), White British, White Irish, any other White background (‘White’) and Not Known (see Supplementary Materials for the breakdown of ethnic groups within each broad ethnic group) [37]. In England, ethnicity data for cancer patients is captured by Trusts at various points in the pathway, and recorded in several datasets including the Cancer Outcomes and Services Dataset (COSD) and Hospital Episode Statistics (HES) database for admitted patient care [38, 39]. Rates were calculated by combining these cancer data with population estimates published by the Office for National Statistics (ONS) [40]; though population estimates by ethnic group are available from other sources they were not suitable for this analysis [41]. Analyses were completed for all cancer sites combined and for 31 specific cancer sites (see Supplementary Materials for ICD-10 codes). Rates were not calculated for the Other (1% of cancer cases) and Not Known ethnic groups (6% of cancer cases); for the former the population was considered too heterogeneous for results to translate to policy, and for the latter no population denominators were available for calculation of rates. Case numbers, rates and ratios for each combination of sex/broad ethnic group/cancer site are reported only when that combination had 100 or more cases altogether in the 5-year study period; rates and ratios are reported only when at least 90% of cases had an ethnic group recorded.

For each cancer site, incidence rates with 95% confidence intervals were calculated for each sex/broad ethnic group combination and standardised to the European 2013 standard population [42]. Rate ratios with 95% confidence intervals were calculated using the White ethnic group as the reference and used to ascertain the statistical significance of differences between each non-White minority ethnic group and the comparable White population. These analyses were completed separately for all age groups combined, people aged 0–64, and people aged 65–90+.

### Sensitivity analyses

To examine the impact of cases with Not Known ethnic group on the rates and ratios calculated in the main analysis, ethnic group was assigned to those cases based on the distribution of the known cases by sex, 5-year age band and cancer site, e.g. cases with Not Known ethnic group were distributed across the Asian, Black, Mixed/Multiple and White ethnic groups in the same proportions as seen in the cases with a known ethnic group. Rates and ratios were re-calculated for all cancer sites, and all cancers combined.

The use of 5 years of cancer data means 5 years of population data is required; however, population data by ethnicity are only captured at the decennial census and are estimated for the following 10 years based on the census data, therefore the necessary population data for this analysis will include some degree of estimation for any five year period. To examine the impact of possible errors in the population estimates on the rates and ratios calculated in the main analysis, a number of plausible perturbations were made to the population data - based on areas of concern raised by the Office for National Statistics (ONS) in their publication of the estimates - and rates and ratios re-calculated for lung, breast, bowel and prostate cancers, and all cancers combined. The sensitivity analyses are described in the Supplementary Materials; in summary they involved changing the ethnic group for a proportion of the population in the oldest and youngest age bands, e.g. increasing the Asian population while decreasing the population in the three other broad ethnic groups.

To examine the plausibility of the main analysis results, rates and ratios for breast, bowel, lung and prostate cancers were calculated for 2012 (using cancer and population data from PHE and ONS as described above) and compared with the 2013–2017 main analysis results. 2012 cancer incidence data have the highest ethnicity data completeness of any year to date, and 2012 population estimates are the closest to the 2011 observed data and are therefore likely to be the most accurate population data in the range of 2012–2017, so together these were considered the best available baseline for the direction and magnitude of differences between ethnic groups.

For all sensitivity analyses, rate ratios with 95% confidence intervals were calculated using the White ethnic group as the reference, and used to ascertain the statistical significance of differences between each non-White minority ethnic group and the comparable White population.

## Results

### Main analysis

The majority of cancer cases in England in 2013–2017 were in the White ethnic group, broadly reflecting the ethnic makeup of the population (Table 1). On average each year in this period there were around 7700 cases in the Asian ethnic group, around 5300 cases in the Black ethnic group, around 1200 cases in the Mixed/Multiple ethnic groups, around 3300 cases in the Other ethnic group and more than 269,000 cases in the White ethnic group. Overall, the number of cases with ethnic group Not Known was larger than the number of cases in any one non-White minority ethnic group. Cervical cancer in situ and non-melanoma skin cancer had high proportions of cases with ethnic groups Not Known, and so were excluded from further analyses.

**Table 1:** Number and percentage of cases by broad ethnic group and cancer site, England, annual average 2013–2017.

	Annual average number (%) of cases in each broad ethnic group					
	White	Asian	Black	Mixed/Multiple	Other	Not Known
All cancers excluding non-melanoma skin cancer	269,450 (89%)	7692 (2.5%)	5281 (1.7%)	1225 (0.4%)	3310 (1.1%)	17,183 (5.6%)
Anal cancer	1096 (93%)	<20 –	<20 –	<20 –	<20 –	43 (3.7%)
Bladder cancer	8077 (93%)	123 (1.4%)	63 (0.7%)	<20 –	67 (0.8%)	347 (4.0%)
Bone sarcoma	393 (82%)	36 (7.4%)	<20 –	<20 –	<20 –	22 (4.5%)
Bowel cancer	31,402 (90%)	719 (2.1%)	488 (1.4%)	109 (0.3%)	341 (1.0%)	1744 (5.0%)
Brain, other CNS and intracranial tumours	8253 (84%)	418 (4.2%)	197 (2.0%)	65 (0.7%)	171 (1.7%)	754 (7.6%)
Breast cancer (females)	39,860 (87%)	1529 (3.3%)	880 (1.9%)	236 (0.5%)	599 (1.3%)	2612 (5.7%)
Breast carcinoma in situ (females)	5641 (83%)	299 (4.4%)	176 (2.6%)	40 (0.6%)	97 (1.4%)	518 (7.7%)
Cancer of unknown primary	6360 (87%)	131 (1.8%)	85 (1.2%)	<20 –	63 (0.9%)	643 (8.8%)
Cervical cancer	2280 (88%)	66 (2.5%)	39 (1.5%)	<20 –	46 (1.8%)	155 (6.0%)
Cervical carcinoma in situ	15,858 (66%)	311 (1.3%)	244 (1.0%)	193 (0.8%)	246 (1.0%)	7129 (30.0%)
Eye cancer	606 (89%)	<20 –	<20 –	<20 –	<20 –	42 (6.1%)
Gallbladder cancer	728 (84%)	53 (6.1%)	24 (2.8%)	<20 –	<20 –	48 (5.6%)
Head and neck cancer	8677 (90%)	336 (3.5%)	120 (1.2%)	40 (0.4%)	106 (1.1%)	388 (4.0%)
Hodgkin lymphoma	1397 (80%)	124 (7.1%)	58 (3.3%)	25 (1.4%)	45 (2.6%)	107 (6.1%)
Kidney cancer	9599 (89%)	284 (2.6%)	183 (1.7%)	41 (0.4%)	117 (1.1%)	590 (5.5%)
Leukaemia	7542 (86%)	300 (3.4%)	149 (1.7%)	53 (0.6%)	122 (1.4%)	565 (6.5%)
Acute lymphoblastic leukaemia	539 (79%)	68 (10.0%)	<20 –	<20 –	21 (3.1%)	<20 –
Acute myeloid leukaemia	2474 (89%)	96 (3.4%)	54 (1.9%)	<20 –	39 (1.4%)	106 (3.8%)
Chronic lymphocytic leukaemia	2935 (86%)	65 (1.9%)	31 (0.9%)	<20 –	32 (0.9%)	335 (9.8%)
Chronic myeloid leukaemia	559 (83%)	40 (5.9%)	<20 –	<20 –	<20 –	41 (6.1%)
Liver cancer	4193 (85%)	237 (4.8%)	97 (2.0%)	20 (0.4%)	70 (1.4%)	287 (5.9%)
Lung cancer	35,304 (92%)	612 (1.6%)	355 (0.9%)	99 (0.3%)	318 (0.8%)	1705 (4.4%)
Melanoma skin cancer	12,120 (91%)	22 (0.2%)	22 (0.2%)	<20 –	66 (0.5%)	1057 (7.9%)
Mesothelioma	2223 (94%)	<20 –	<20 –	<20 –	<20 –	95 (4.0%)
Myeloma	4239 (86%)	157 (3.2%)	223 (4.5%)	29 (0.6%)	62 (1.3%)	220 (4.5%)
Non-Hodgkin lymphoma	10,458 (88%)	433 (3.6%)	195 (1.6%)	60 (0.5%)	155 (1.3%)	631 (5.3%)
Non-melanoma skin cancer^a^	98,388 (80%)	190 (0.2%)	93 (0.1%)	92 (0.1%)	457 (0.4%)	24,537 (20.0%)
Oesophageal cancer	7005 (93%)	102 (1.4%)	59 (0.8%)	<20 –	47 (0.6%)	278 (3.7%)
Ovarian cancer	5449 (87%)	222 (3.5%)	88 (1.4%)	29 (0.5%)	76 (1.2%)	404 (6.4%)
Pancreatic cancer	7539 (89%)	181 (2.1%)	154 (1.8%)	32 (0.4%)	87 (1.0%)	505 (5.9%)
Penile cancer	480 (91%)	<20 –	<20 –	<20 –	<20 –	24 (4.5%)
Prostate cancer	35,782 (87%)	732 (1.8%)	1293 (3.1%)	160 (0.4%)	381 (0.9%)	2887 (7.0%)
Small intestine cancer	1208 (89%)	42 (3.1%)	29 (2.1%)	<20 –	<20 –	63 (4.7%)
Stomach cancer	4788 (88%)	164 (3.0%)	149 (2.7%)	26 (0.5%)	68 (1.2%)	238 (4.4%)
Testicular cancer	1633 (83%)	73 (3.8%)	<20 –	<20 –	43 (2.2%)	183 (9.3%)
Thyroid cancer	2352 (77%)	273 (8.9%)	88 (2.9%)	29 (0.9%)	91 (3.0%)	223 (7.3%)
Uterine cancer	6663 (86%)	316 (4.1%)	172 (2.2%)	38 (0.5%)	94 (1.2%)	436 (5.6%)
Vaginal cancer	184 (91%)	<20 –	<20 –	<20 –	<20 –	<20 –
Vulval cancer	1001 (92%)	<20 –	<20 –	<20 –	<20 –	43 (4.0%)
*Population (millions)*						
* Female Population 0–64*	18.49 (82.4%)	2.06 (9.2%)	0.96 (4.3%)	0.68 (3.0%)	0.25 (1.1%)	*N/A* *N/A*
* Female Population 65–90*+	5.04 (95.1%)	0.15 (2.9%)	0.07 (1.3%)	0.02 (0.4%)	0.02 (0.3%)	*N/A* *N/A*
* Female Population 0–90*+	23.54 (84.8%)	2.21 (8.0%)	1.03 (3.7%)	0.70 (2.5%)	0.27 (1.0%)	*N/A* *N/A*
* Male Population 0–64*	18.62 (82.3%)	2.11 (9.3%)	0.91 (4.0%)	0.68 (3.0%)	0.31 (1.4%)	*N/A* *N/A*
* Male Population 65–90*+	4.17 (94.9%)	0.13 (3.1%)	0.05 (1.2%)	0.02 (0.4%)	0.02 (0.4%)	*N/A* *N/A*
* Male Population 0–90*+	22.78 (84.3%)	2.25 (8.3%)	0.96 (3.6%)	0.70 (2.6%)	0.33 (1.2%)	*N/A* *N/A*

Case numbers and percentages not reported for sex/broad ethnic group combinations with fewer than 100 cases over the 5-year study period (e.g. annual average fewer than 20 cases).

^a^There is known under-recording of NMSC so numbers may be an underestimate.

In all broad ethnic groups, lung, bowel, breast and prostate cancers were the four most common cancer sites; breast cancer was the most common site in all groups except for the Black ethnic group, where prostate cancer was the most common, and bowel cancer was more common than lung cancer in all groups except for the White ethnic group. Beyond this there was some variation by broad ethnic group, for example, melanoma skin cancer was the fifth most common cancer site for the White ethnic group but was not in the 20 most common cancers for the Asian, Black or Mixed/Multiple ethnic groups. Uterine cancer was in the 10 most common cancers for the Asian, Black and Mixed/Multiple ethnic groups but only 14th most common for the White ethnic group.

**Fig. 1:**
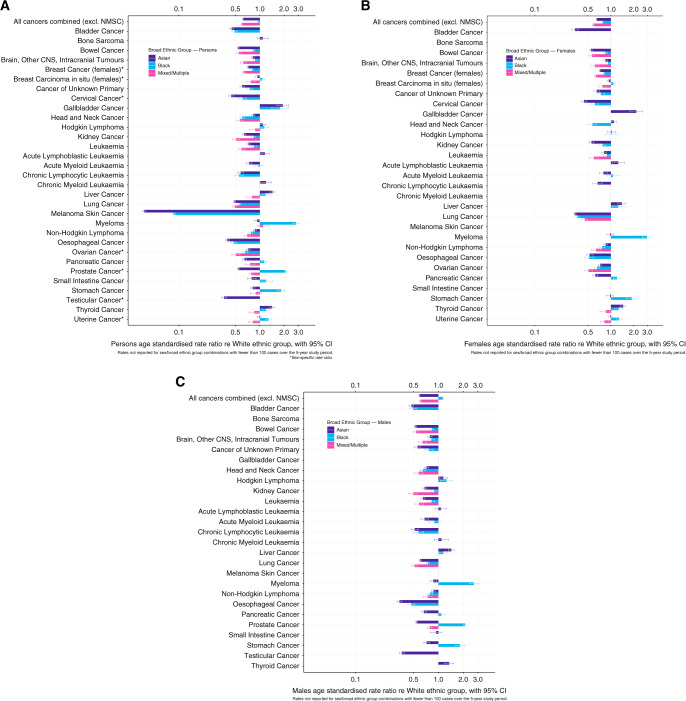
Rate ratios not reported for cancer site/sex/broad ethnic group combinations with fewer than 100 cases over the 5-year study period. *Sex-specific rate ratio. Rate ratios of age-standardised incidence rates for persons (**A**), females (**B**) and males (**C**), by cancer site, for Asian, Black and Mixed/Multiple ethnic groups with reference to the White ethnic group.

Figure 1A–C and Supplementary Materials show that for most cancer sites, rates in the Asian, Black and Mixed/Multiple ethnic groups for females and males were significantly lower than in the White group. For most cancer site/sex/broad ethnic group combinations, rate ratios for people aged 0–64 and 65–90+ separately were in the same direction and of similar magnitude to the all-ages combined analysis. Rate ratios greater than 1 (indicating higher rates compared with the White ethnic group) were seen most frequently in the Black ethnic group, people aged 0–64, and for gastrointestinal cancer sites, excluding bowel cancer.

For all cancers combined (excluding non-melanoma skin cancer), age-standardised incidence rates in non-White minority ethnic groups were significantly lower than in the White ethnic group, except for Black males where rates were 14% higher compared with White males. This difference was driven by prostate cancer, the rate of which was 2.1 times higher in the Black ethnic group compared with the White ethnic group, and which contributes a high proportion of all cancers combined total cases.

The highest rate ratios were for myeloma in the Black ethnic group, with rates 2.7–3.0 times higher in this population compared with the corresponding White ethnic group. Several gastrointestinal cancer sites (gallbladder, liver, pancreatic and stomach), plus Hodgkin lymphoma, thyroid and uterine cancers, had higher incidence rates in the Black ethnic group compared with the White ethnic group. Gallbladder, Hodgkin lymphoma, liver and thyroid cancers also had higher incidence rates in the Asian ethnic group compared with the White ethnic group.

For all cancers combined, differences compared with the White group for Asian and Mixed/Multiple persons were smaller in those aged 65–90+ than in those aged 0–64. For most cancer sites with a sufficient number of cases to explore by age, rate ratios were in the same direction in both the 0–64 and 65–90+ age groups, but of a larger magnitude in the younger age group (see Supplementary Materials). Prostate cancer incidence in Black compared with White males was 2.9 times higher in those aged 0–64, and 1.9 times higher in those aged 65–90+. Liver cancer incidence in Black compared with White males was 1.6 times higher in those aged 0–64 but around the same in those aged 65–90+. There were a small number of cancer site/broad ethnic group combinations in women where the direction of the rate ratio differed between the two age groups, but the rate ratios themselves were relatively small, in line with other rate ratios found.

Several anogenital cancers had too few cases in non-White minority ethnic groups for results of every sex and ethnic group combination to be calculated, as did some overall less common cancer sites including eye cancer and mesothelioma, and some common cancer sites with particularly low incidence in non-White minority ethnic groups such as melanoma skin cancer. In the analysis split by age groups, many cancer sites/broad ethnic group/sex combinations had too few cases for results to be calculated. The Mixed/Multiple ethnic groups had the most cancer sites with case numbers too low for results to be calculated, reflecting the overall younger age profile of this ethnic group.

### Sensitivity analyses

Results tables and figures for the sensitivity analyses are presented in Supplementary Materials.

Assigning Asian, Black, Mixed/Multiple, White or Other ethnic groups for cases with ethnic group Not Known, based on the distribution of ethnic groups in cases with known ethnicity, had very little effect on the direction, significance and magnitude of differences between non-White minority ethnic groups and the White ethnic group. Rate ratios changed by a maximum of two percentage points compared with the main analysis for all cancer sites. Significance and direction of rate ratios remained unchanged for 171 of the 172 combinations of sex/broad ethnic group/cancer site; rates of small intestine cancer in Black persons became significantly higher than the corresponding White ethnic group in this analysis (previously statistically similar).

Changing ethnic group in the population for the youngest and oldest age bands (from the White ethnic group to the non-White minority ethnic groups; ‘White population decrease’ in Supplementary Materials) and changing ethnic group in the population in all age bands (from the non-White minority ethnic groups to the White ethnic group, ‘White population increase’), or increasing the Asian population and decreasing the other ethnic group populations overall (‘Asian population increase’), gave the same direction and significance of differences as the main analysis, for most combinations of sex/broad ethnic group/cancer site in the five cancer sites examined (all cancer sites combined, bowel, female breast, lung, and prostate). Rate ratios changed by a maximum of 5 percentage points in the Asian and Mixed/Multiple ethnic groups, and 12 percentage points in the Black ethnic group.

For all cancer sites combined, bowel, female breast, lung and prostate cancers, rate ratios were in the same direction and of similar magnitude in the 2012 data and the 2013–2017 data.

## Discussion

### Summary of results

Incidence rates for all cancers combined were significantly lower for the non-White minority ethnic groups compared with the White ethnic group except for Black males, where rates were higher compared with White males, driven by the prostate cancer incidence rate which was twice as high for Black males as for White males.

For most specific cancer sites, the incidence was lower or similar in non-White minority ethnic groups compared with the White ethnic group, with some exceptions: rates of prostate, gallbladder, liver, pancreatic, stomach, thyroid and uterine cancers, myeloma, and Hodgkin lymphoma, were higher in one or more non-White minority ethnic groups with reference to the comparable White ethnic group population. Rate ratios were highest for myeloma in the Black ethnic group, with rates almost threefold higher than those of the comparable White population. Analysis by age showed that higher rates in non-White minority ethnic groups compared with the White ethnic group were often more pronounced in people aged under 65.

Low numbers of cases precluded some analyses particularly in the Mixed/Multiple ethnic groups. Sensitivity analyses demonstrated that the direction, significance and magnitude of differences between non-White minority ethnic groups were largely unaffected by the assignment of an ethnic group for the small proportion of cases without a recorded ethnic group, or by feasible errors in the population estimates used to calculate the age-standardised rates. This indicates the feasible margins of error around ethnicity in cancer and population data are smaller than the actual differences in incidence rates between broad non-White minority ethnic groups and the White ethnic group.

### Comparison with existing evidence

These results largely reflect patterns previously reported, though there are some differences. The findings around most cancer sites corroborate and expand upon previous analyses for England and London [6–11, 43], but run counter to some results from Scotland, Sweden, Denmark and the Netherlands [4, 44]. These differences between nations may reflect many factors: tenure/generation/makeup of the non-White minority ethnicity populations included, and cancer risk in the indigenous population—both of which relate to risk factor prevalence and access to healthcare e.g. prostate-specific antigen (PSA) testing—as well as methodology and time period of the studies.

### Possible mechanisms for differences in cancer incidence between ethnic groups

A review of evidence from industrialised European countries published between 1990 and 2010 grouped cancer sites by their risk factors and incidence rates in different populations. Cancer sites related to infections—including liver, stomach and cervical cancers—typically have a higher incidence in non-White minority ethnic groups compared with the White population [4]. Cancer sites related to aspects of ‘western lifestyle’ like smoking, reproductive behaviours, excess body weight and diet—including bowel, breast and lung cancers—have a lower incidence in non-White minority ethnic groups [4]. These broad themes are partly borne out in the present analysis—the latter more consistently than the former—and they reflect patterns of risk factor exposure in England. The higher prevalence of hepatitis and *H. pylori* infections compared with the general population in some non-White minority ethnic groups is reflected in their higher rates of liver and stomach cancers [45–49]. Lower or similar smoking prevalence compared with the general population is evident in all Black and Asian subgroups except Pakistani and Bangladeshi men. Particularly low smoking prevalence is observed in Asian and Black African women, although small proportions of Asian men and women use chewing tobacco as well as, or instead of, cigarettes. These observed differences in smoking prevalence are reflected in low rates of lung and head and neck cancers [50]. Lower prevalence of overweight and obesity compared with the general population in all Black and Asian subgroups except Black Caribbean men and women, Black African women, and Pakistani women is congruent with their lower rates of breast, bowel and uterine cancers [50]; their lower screening uptake and different reproductive behaviour is also likely relevant for some of these cancer sites [12–14, 51].

Some cancer sites though show notable deviation from this expected pattern. Cervical cancer is caused by persistent Human Papillomavirus (HPV) infection, but the incidence was lower in Asian and Black women compared with White women aged 0–64 (which also drove lower incidence in all-ages combined). Evidence on cervical cancer incidence by ethnic group is relatively mixed [2, 4, 8], probably reflecting the complex interplay between HPV infection prevalence and cervical screening uptake, both of which may vary within ethnic groups over time, between subgroups and across adopted home nations. Thyroid cancer, which is associated with being overweight or obese, had a higher incidence in the Asian ethnic group than in the White ethnic group; however, known risk factors may be unlikely to explain these ethnic differences [9].

Exogenous risk factors are likely to explain the bulk of the difference in cancer incidence rates between ethnic groups, as germline genetic mutations contribute only a small proportion of cases overall [52], and as the phenotypic characteristics of different ethnic groups overlap substantially. This may vary between cancer sites and risk factors: lower breast cancer rates in Asian women in England may be fully explained in terms of known risk factors [53], while ethnic differences in lung cancer incidence among female never-smokers in England persist after comprehensive adjustment for risk factors [54].

In the England population overall, age-standardised cancer incidence is positively associated with socioeconomic deprivation, and this is largely attributable to the higher prevalence of key cancer risk factors in more deprived groups [50, 55, 56]. Deprivation and non-White minority ethnicity are also highly correlated in England [57], but as described above this generally does not translate to higher risk factor prevalence in these populations. Currently, lower prevalence of the most harmful cancer risk factors, and perhaps the ‘healthy migrant effect’ (migrant populations often being in better health than the indigenous population in their new home country), largely outweighs the harmful effect of deprivation to provide a ‘cancer protective’ effect of non-White minority ethnicity [4, 58, 59]. But there is evidence that as minority ethnic groups become more established in their adopted home nations, their risk factor prevalence and corresponding cancer incidence rates become increasingly similar to those of the indigenous population [60–62]. This coupled with overall higher rates of deprivation in non-White minority ethnic groups could in future mean the association between deprivation and cancer incidence is actually compounded in these groups.

### Strengths and limitations

This work updates evidence in an important area with clear implications for policy and practice, and is particularly timely given the renewed focus on the health of minority ethnic groups in the UK during the COVID-19 pandemic where these groups were disproportionately affected [63]. The most recent analyses of cancer incidence by ethnic group in England were for data in 2002–2006 and 2001–2007, when ethnicity recording in cancer data was relatively poor; this new analysis capitalises on substantial improvements in ethnicity data collection since 2012. Using 5 years of data provides a large number of cases, which increases confidence in the results. Using a consistent method across cancer sites, ethnic groups and sexes facilitates comparison between groups. For some cancer sites this is the first time the numbers of cancer cases in each ethnic group have been published in England, and for all cancer sites the number of cases in each ethnic group has the highest degree of certainty to date.

There are, however, limitations to this study. Analysing broad rather than specific ethnic groups masks known variation between those specific groups [5–11, 27], though it affords more statistical power through larger numbers of cases. Imputation of missing ethnicity in cancer data based on observed cases assumes ethnicity is missing at random and therefore by definition does not allow for any variation by ethnic group in the likelihood of having ethnicity recorded. Such variation could affect the incidence rates observed here; however, evidence is lacking on how to more accurately account for missing ethnicity. Name analysis was not possible with the de-identified data used here, and may be unreliable for some ethnic groups, but this approach could be considered for further analyses in this area [64, 65]. Ethnicity recording in NHS data may be less accurate for minority ethnicity populations than the White British population [66, 67], and further varies by characteristics including age, sex, geographical region, and care pathway [68], although it is unclear how much of a concern this is for the recording of broad ethnic groups in the data period studied here. Other studies have used different theoretical scenarios e.g. assuming White people are less likely to have an ethnic group recorded or vice versa, but have obtained incidence rate ratios of similar magnitude to those in the present analysis, indicating differences in cancer incidence between broad ethnic groups are sufficiently large to outweigh potential data biases, especially when data completeness is relatively high. The population estimates used in these calculations are subject to uncertainty in several areas as they do not account for migration or differences in life expectancy and birth rate by ethnic group; however, sensitivity analyses show the direction, significance and magnitude of the main analysis results are robust to feasible margins of error in the population data, and presently no better population estimates are available for the age bands and data years required for age-standardised cancer incidence rate calculations. One alternative population data source, Ethpop, has taken international migration patterns into account but covers only two of the five years included in the current analysis (2015 and 2016). Ethpop’s relative proportion of each broad ethnic group is very similar to the ONS data in the age groups most relevant for cancer analysis, so using this data source would likely result in similar ASR ratios to those reported here.

Though ‘gold standard’ population data will be available with the 2021 census, the low number of cancer cases in some combinations of sex/broad ethnic group/cancer sites requires the use of multiple years of cancer data to ensure robust results, so some level of estimation around the census year population data will always be needed. Repeating the analysis with population data from the 2021 census and 2021 cancer incidence (possibly only for more common cancer sites, to ensure sufficient numbers of cases) will be important for further assessing the robustness of the present results; however, the likely impact of the pandemic on cancer data for 2021 and beyond must be borne in mind, these results may not be generalisable.

### Future policy and research directions

Although incidence rates are lower and survival may be higher in some ethnic groups for some cancer sites [2, 24, 25], people from non-White minority ethnic groups typically rate their cancer patient experience less positively [29], and their cancer may be diagnosed at a later stage, compared with people in the White ethnic group [20, 69]. People from non-White minority ethnic groups may also have lower awareness of cancer risk factors and symptoms compared with people in the White ethnic group, and longer diagnostic intervals (which may reflect lower awareness on the part of patients or clinicians), with possible variation by cancer site [69–71]. Interventions to improve awareness of cancer risk factors, uptake of screening, knowledge of cancer signs and symptoms [72], and medication adherence can be modified to be more effective for people in minority ethnic groups [73–75]; principles of these interventions could be used more widely. People of non-White minority ethnicity may be less likely to be recruited into clinical trials [76]. Underpinning the successful design, implementation and evaluation of interventions to improve the experience and outcomes of cancer for patients of non-White minority ethnicity is data availability, and the current analysis has highlighted gaps (despite substantial improvements in the past decade) in capturing ethnicity information in cancer data, population estimates, and risk factor prevalence surveys. Improving the collection of ethnicity data in the NHS and recording ethnicity on death certificates could increase the accuracy of cancer incidence and survival data for each ethnic group. Such improvement would require substantial engagement with professionals and the public, not least building trust that information on ethnicity is only collected to help determine health differences, reduce inequalities and facilitate further research and service improvement. For professionals, recommendations for improving ethnicity recording in health service datasets include routine quality assessment/review from internal and external bodies, particularly around non-specific codes e.g. ‘any other ethnic group’; better use of ethnicity data by healthcare researchers, including an appropriate critique of the source data; and improved guidance from healthcare leaders around collection and recording of ethnicity data ‘on the ground’, with sufficient monitoring of the implementation of that guidance [68].

## Conclusion

Though a small number of cancer sites have higher incidence rates in Asian, Black and Mixed/Multiple ethnic groups, for the majority of cancer sites these groups have a lower incidence than the White population. Differing prevalence of risk factors and access to/use of health services is likely to explain more of this variation than are genetic factors; if risk factor prevalence changes cancer rates may rise in minority ethnic groups, therefore action to address key risk factors and to improve the cancer experiences and outcomes of people in minority ethnic groups is vital. Improving the collection of ethnicity information in healthcare datasets will support a better understanding of differences in disease, as well as inequalities in cancer and where improvements in the health service can be made.

## Supplementary information

Reproducibility Checklist

Dataset 1

## Data Availability

The cancer incidence data analysed during the current study are available from the National Cancer Registration and Analysis Service (part of Public Health England), on request through the Office for Data Release (see methods) but restrictions apply to the availability of these data, which were used under license for the current study and so are not publicly available. The population data are publicly available (see methods).

## Code availability

Age-standardised rates, rate ratios and sensitivity analysis were calculated using standard methods therefore the code is not provided.
